# Women’s addiction : experiences at Arrazi Psychiatric Hospital in Morocco

**DOI:** 10.1192/j.eurpsy.2024.822

**Published:** 2024-08-27

**Authors:** A. Khallouk

**Affiliations:** Arrazi Psychiatric Hospital, salé, Morocco

## Abstract

**Introduction:**

Drug addiction, also called substance use disorder, is a disease that affects a person’s brain and behavior and leads to an inability to control the use of a legal or illegal drug or medicine. there is a complex interplay of neurobiology, genetics, and the environment --nature and nurture-- that play into the development of addiction, alcohol, and other drug use disorder.Substances such as alcohol, marijuana and nicotine also are considered drugs.

Research has shown that women often use drugs differently, respond to drugs differently, andcan have unique obstacles to effective treatment as simple as not being able to find child care orbeing prescribed treatment that has not been adequately tested on women.

**Objectives:**

describe the socio-demographic and clinical characteristics of female patients admitted to the addictology department of Arrazi Hospital in Salé

**Methods:**

Retrospective study with descriptive and analytical aims on the files of women who were admitted to the addictology service since its opening in 2000, with the aim of specifying the prevalence and the characteristics of addictive behaviors in the female population.

**Results:**

in progress

**Conclusions:**

in progress

**Disclosure of Interest:**

None Declared

